# Pestalafuranones F–J, Five New Furanone Analogues from the Endophytic Fungus *Nigrospora* sp. BM-2

**DOI:** 10.3390/molecules19010819

**Published:** 2014-01-10

**Authors:** Hongqi Zhang, Zhangshuang Deng, Zhiyong Guo, Xuan Tu, Junzhi Wang, Kun Zou

**Affiliations:** Hubei Key Laboratory of Natural Products Research and Development, College of Chemistry and Life Science, China Three Gorges University, Yichang 443002, China

**Keywords:** pestalafuranones F–J, endophytic fungus, *Nigrospora* sp., cytotoxic activity

## Abstract

Five new 2(*5H*)-furanone-type derivatives, pestalafuranones F–J (compounds **3**–**7**), together with two known compounds, pestalafuranones A (**1**) and B (**2**), were isolated from the ethyl acetate extract from the fermentation broth of the endophytic fungus *Nigrospora* sp. BM-2 in a hypersaline medium. The structures of these metabolites were elucidated by EIMS, HREIMS and NMR spectroscopic data. Compounds **1**–**7** exhibited no cytotoxic activities against the MDA-MB-231 and Caski cancer cell lines.

## 1. Introduction

It has been reported that the extreme or dystrophic environments in which microorganisms grow might disturb the biosynthetic pathways of their secondary metabolites [1,2]. As a strategy for obtaining structurally novel and bioactive compounds, hypersaline growth medium is usually employed when cultivating fungi [3,4,5]. In our previous unpublished studies, pyrone, ergosterol, coumarin and anthraquionone derivatives were identified as secondary metabolites when potato dextrose broth was used to grow the endophytic fungus *Nigrospora* sp*.* BM-2 isolated from *Saccharum arundinaceum* Retz. [6]. Recently, seven 2(*5H*)-furanone-type derivatives never isolated from potato dextrose broth were obtained from modified medium with addition 3% NaCl. Herein, we report the detailed isolation, structural characterization, and cytotoxic activity of pestalafuranones F–J (compounds **3**–**7**).

## 2. Results and Discussion

### 2.1. Structural Characterization

The known compounds pestalafuranone A (**1**) and B (**2**) were obtained, respectively, as colorless powders with the same molecular formula C_11_H_14_O_3_. Their chemical structures were identified by comparison of the ^1^H and ^13^C-NMR data with published results [7].

Pestalafuranone F (**3**) was isolated as a pale yellow oil. The molecular formula was determined to be C_11_H_14_O_2_ according to a HREIMS peak at *m/z* 178.0989 [M]^+^ (calcd. 178.0994). Compared to **2** [C_11_H_14_O_3_], a loss of oxygen was deduced. Further comparison of the ^1^H and ^13^C-NMR data (Table 1 and Table 2) exhibited two noteworthy highfield shifts of the H-11 proton (*δ*_H_ 2.28 ppm, Δ −2.25 ppm) and C-11 carbon (*δ*_C_ 26.6 ppm, Δ −41.4 ppm). These data suggested that the oxymethine (*δ*_H_ 4.53 ppm and *δ*_C_ 68.0 ppm) of **2** was alkylated to give a methylene unit, leading to the assignment of the structure of **3**.

**Table 1:** ^1^H-NMR Data for compounds **3**–**7** in CDCl_3_ (400 MHz, *δ* ppm, *J* in Hz).

No.	3	4	5	6	7
5	4.85, s	4.69, s	4.89, s	4.89, d (3.5)	4.91, s
6	6.23, dd (1.6, 16.0)	6.12, dd (1.0, 15.8)	6.51, dt (1.8, 16.0)	6.08, dd (1.5, 15.7)	6.28, dd (1.5, 16.0)
7	6.92, m	6.87, m	7.08, m	6.97, m	6.99, m
8	1.89, d (6.8)	1.87, d (6.6)	4.36, d (4.8)	1.86, d (6.3)	1.94, d (6.8)
9	6.54, d (16.0)	2.61, m	6.59, d (16.0)	4.87, d (4.0)	6.70, d (16.0)
10	6.07, dt (6.6, 16.0)	1.64, m	6.13, dt (6.6, 16.0)	3.87, dt (4.0, 8.1)	6.08, dt (7.2, 16.0)
11	2.28, m	3.84, dt (6.2, 12.3)	2.30, m	1.40, m	2.57, q (6.1)
12	1.10, t (7.4)	1.26, dd (0.6, 6.8)	1.10, t (7.4)	1.00, t (7.4)	3.84, t (6.1)

**Table 2:** ^13^C-NMR Data for compounds **3**–**7** in CDCl_3_ (100 MHz, *δ* ppm).

No.	3	4	5	6	7
2	173.3, qC	173.2, qC	173.1, qC	172.7, qC	173.1, qC
3	120.7, qC	122.9, qC	119.8, qC	124.3, qC	121.4, qC
4	150.6, qC	158.1, qC	152.6, qC	155.4, qC	149.8, qC
5	68.5, CH_2_	70.7, CH_2_	68.6, CH_2_	69.8, CH_2_	68.5, CH_2_
6	118.6, CH	118.3, CH	116.7, CH	118.1, CH	118.5, CH
7	133.3, CH	132.6, CH	135.1, CH	134.3, CH	133.8, CH
8	19.4, CH_3_	19.3, CH_3_	63.4, CH_2_	19.3, CH_3_	19.4, CH_3_
9	119.5, CH	23.2, CH_2_	119.3, CH	70.1, CH	122.6, CH
10	140.8, CH	36.7, CH_2_	141.9, CH	75.4, CH	135.0, CH
11	26.6, CH_2_	67.3, CH	26.6, CH_2_	25.0, CH_2_	36.7, CH_2_
12	12.9, CH_3_	23.9, CH_3_	12.8, CH_3_	10.2, CH_3_	61.5, CH_2_

Pestalafuranone G (**4**) with the molecular formula C_11_H_16_O_3_ from HREIMS (*m/z* 196.1099 [M]^+^, calcd. 196.1095) was obtained as a pale yellow oil. Its ^1^H-NMR spectrum (Table 1) was similar to that of **2** except that signals for two olefinic methines (*δ*_H_ 6.77 and 6.03 ppm) were replaced with two methylene signals (*δ*_H_ 2.61 and 1.64 ppm) of an ethane units, which was further supported by two highfield shifts of the C-9 and C-10 carbon (*δ*_C_ 23.2 and 36.7 ppm). The structural changes of two units at H-9/H-10 and C-9/C-10 were confirmed by HMBC and ^1^H-^1^H COSY correlations. Consequently, pestalafuranone G (**4**) was identified as a reduced derivative of **2**.

**Figure 1:**
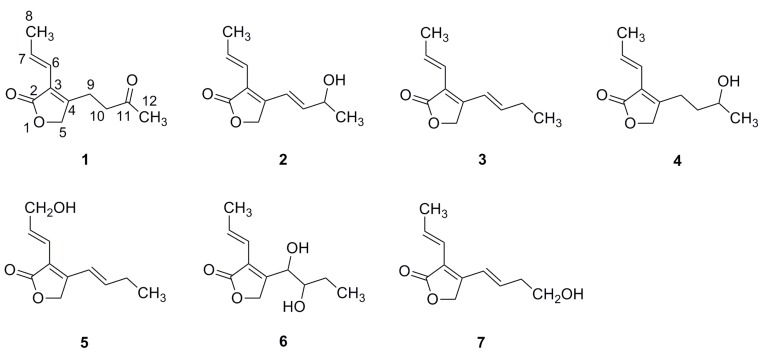
Structures of compounds **1**–**7**.

Pestalafuranone H (**5**) afforded colorless needles and its formula was determined to be C_11_H_14_O_3_ based on the molecular ion peak at *m/z* 194.0938 [M]^+^ (calcd 194.0943) in the HREIMS spectrum, with one oxygen atom more than that of **3**. Its 1D NMR spectral data (Table 1 and Table 2) were similar to those of **3** except for the lack of a methyl group, an additional oxygenated methylene group, and downfield shifts for C-8. These data indicated that **5** was a derivative of **3** hydroxylated on the 8-methyl group, which was also supported by 2D NMR spectra. Thus, the structure of **5** was elucidated as shown in Figure 1.

Pestalafuranone I (**6**) was purified as a pale yellow oil. Its HREIMS exhibits a peak at *m/z* 212.1042 [M]^+^ (calcd. 212.1049), indicating a molecular formula C_11_H_16_O_4_. The ^1^H and ^13^C NMR spectra of **6** (Table 1 and Table 2) suggested the presence of the same furanone ring with a propenyl group attached to C-3, as that appearing in compounds **1**–**4**. The ^1^H-^1^H COSY and HMBC correlations indicated a *n*-butyl subunit (C9-C12) in the molecule, and the linkage between C-4 of the furanone ring and C-9 of the *n*-butyl side chain was established according to the HMBC correlations of H-9 (*δ*_H_ 4.87 ppm) with C-3 (*δ*_C_ 124.3 ppm) and C-4 (*δ*_C_ 155.4 ppm). Furthermore, the ^13^C-NMR chemical shifts of C-9 (*δ*_C_ 70.1 ppm) and C-10 (*δ*_C_ 75.4 ppm) were explained by the introduction of two hydroxyl groups, as demonstrated by a molecular weight 34 amu greater than **3**. Finally, assignment for planar structure of **6** was based on analogy to **3** and comprehensive NMR experiments.

Pestalafuranone J (**7**) was obtained as a pale yellow oil with a molecular formula identical to that of **5** (HREIMS *m/z* 194.0938 [M]^+^, calcd. 194.0943), indicating the presence of a hydroxyl group. Compared to **3**, the ^1^H-NMR spectrum (Table 1) displayed that the methyl signal (*δ*_H_ 1.26 ppm) was replaced by oxygenated methylene signal (*δ*_H_ 3.84 ppm). Furthermore, the HMBC correlations of the oxygenated methylene proton (*δ*_H_ 3.84 ppm) in **7** with C-10 suggested that a hydroxyl group was attached to C-12 position as depicted in **7**. Thus, **7** differed from **5** in the location of the hydroxyl group in the *n*-butyl skeleton.

### 2.2. Cytotoxic Activities

The isolates **1**–**7** were evaluated for cytotoxicity against MDA-MB-231 and Caski cancer cell lines using the MTT method and they were shown to be inactive.

## 3. Experimental

### 3.1. General

NMR spectra were measured on a Bruker Ultrashied TM Plus 400 MHz spectrometer in chloroform-d as a solvent with tetramethylsilane as an internal standard and chemical shifts were recorded in *δ* values. EI and HREIMS spectra were recorded on DSQ II and MAT95XP mass spectrometer respectively. UV spectra were taken on UV-3100PC spectrometer. Optical rotations were measured on a WZZ-2S polarimeter.

### 3.2. Fungal Material

The endophytic fungus *Nigrospora* sp. BM2 was isolated from a piece of fresh tissue from the inner part of a medicinal plant leaf of *Saccharum arundinaceum* Retz., collected from Yichang (Hubei Province, China) in April 2011. The fungus was deposited as *Nigrospora* sp. BM2 (GenBank accession numbers JN687964) at Hubei Key Laboratory of Natural Products Research and Development, College of Chemistry and Life Sciences, China Three Gorges University, Yichang, China.

*Nigrospora* sp. BM2 was maintained on potato dextrose agar. Agar plugs, containing the fungal strain, were inoculated in 500 mL Erlenmeyer flasks, each containing 200 mL of potato dextrose broth. Flask cultures were incubated at 28 °C on a rotary shaker at 130 rpm for 3 days as seed culture. Each of the seed cultures (200 mL) was transferred into 500 mL Erlenmeyer flasks containing 200 mL of potato dextrose broth supplemented with 3% NaCl. These flasks were incubated at 28 °C on a rotary shaker at 130 rpm for 10 days. After fermentation, the culture (total volume 10 L) was centrifuged to yield the supernatant and a mycelial cake. The supernatant was extracted with an equal volume of EtOAc three times, the extracts were combined and solvent was removed under reduced pressure. The mycelial cake was immersed in 1 L of acetone, the organic layers were collected and removed under reduced pressure. The two residues (5.8 g) were combined for isolation.

### 3.3. Extraction and Isolation

The residues was chromatographed on a Sephadex LH-20 column eluted with CHCl_3_/MeOH (v/v = 1/1) to yield five fractions (Fr. 1-Fr. 5). Fr. 3 (98 mg) was further separated by semipreparative reversed-phase HPLC on a ODS semipreparative C18 column (COSMOSIL 5 μm, 10 × 250 mm) eluted with 30% MeCN/H_2_O to afford pestalafuranones B (**1**, 20 mg) and G (**4**, 10 mg). Fr. 4 (50 mg) was successively subjected to semipreparative reversed-phase HPLC eluted with 35% MeCN/H_2_O to afford pestalafuranones H (**5**, 2 mg), I (**6**, 3 mg), J (**7**, 2 mg) and A (**2**, 2mg). Fr. 5 (40 mg) was further purified by semipreparative reversed-phase HPLC eluted with 75% MeCN/H_2_O to afford pestalafuranone F (**3**, 3 mg).

*Pestalafuranone A* (**1**). Colorless powder; UV (CH_3_OH) λ_max_ 247 (ε 6480) nm; EIMS *m/z* 194 [M]^+^ (calcd for C_11_H_14_O_3_, 194). ^1^H-NMR (400 MHz, CDCl_3_): 6.92 (1H, m, H-7), 6.14 (1H, dd, *J* = 1.6 and 15.8 Hz, H-6), 4.70 (2H, s, H-5), 2.76 (4H, m, H-9 and H-10), 2.23 (3H, s, H-12), 1.91 (3H, d, *J* = 6.8 Hz, H-8); ^13^C-NMR (100 MHz, CDCl_3_): 206.1 (qC, C-11), 172.8 (qC, C-2), 156.6 (qC, C-4), 133.1 (CH, C-7), 123.4 (qC, C-3), 118.1 (CH, C-6), 70.8 (CH_2_, C-5), 41.0 (CH_2_, C-10), 29.8 (CH_3_, C-12), 20.4 (CH_2_, C-9), 19.3 (CH_3_, C-8).

*Pestalafuranone B* (**2**). Colorless powder; UV (CH_3_OH) λ_max_ 245 (ε 6360) nm; [α]_D_ −49.5 (c 0.1, CH_3_OH); EIMS *m/z* 194 [M]^+^ (calcd for C_11_H_14_O_3_, 194). ^1^H-NMR (400 MHz, CDCl_3_): 6.95 (1H, m, H-7), 6.77 (1H, d, *J* = 16.0 Hz, H-9), 6.26 (1H, dd, *J* = 1.6 and 15.7 Hz, H-6), 6.03 (1H, dd, *J* = 5.3 and 16.0 Hz, H-10), 4.86 (2H, s, H-5), 4.53 (1H, m, H-11), 1.90 (3H, d, *J* = 6.8 Hz, H-8), 1.37 (3H, d, *J* = 6.5 Hz, H-12); ^13^C-NMR (100 MHz, CDCl_3_): 173.1 (qC, C-2), 149.4 (qC, C-4), 140.9 (CH, C-10), 134.1 (CH, C-7), 122.3 (qC, C-3), 118.41 (CH, C-6), 118.36 (CH, C-9), 68.4 (CH_2_, C-5), 68.0 (CH, C-11), 23.2 (CH_3_, C-12), 19.4 (CH_3_, C-8).

*Pestalafuranone F* (**3**). Pale yellow oil; UV (CH_3_OH) λ_max_ 290 (ε 6500), 220 (ε 4600) nm; ^1^H- and ^13^C-NMR data, see Table 1 and Table 2; HREIMS *m/z* 178.0989 [M]^+^ (calcd for C_11_H_14_O_2_, 178.0994).

*Pestalafuranone G* (**4**). Pale yellow oil; UV (CH_3_OH) λ_max_ 245 (ε 6300) nm; [α]_D_ −56.0 (c 0.1, CH_3_OH); ^1^H- and ^13^C-NMR data, see Table 1 and Table 2; HREIMS *m/z* 196.1095 [M]^+^ (calcd for C_11_H_16_O_3_, 196.1099). 

*Pestalafuranone H* (**5**). Colorless needles; UV (CH_3_OH) λ_max_ 290 (ε 6360), 224 (ε 4320) nm; ^1^H- and ^13^C-NMR data, see Table 1 and Table 2; HREIMS *m/z* 194.0938 [M]^+^ (calcd for C_11_H_14_O_3_, 194.0943).

*Pestalafuranone I* (**6**). Pale yellow oil; UV (CH_3_OH) λ_max_ 249 (ε 6480) nm; [α]_D_ +27.2 (c 0.1, CH_3_OH); ^1^H- and ^13^C-NMR data, see Table 1 and Table 2; HREIMS *m/z* 212.1042 [M]^+^ (calcd for C_11_H_16_O_4_, 212.1049).

*Pestalafuranone J* (**7**). Pale yellow oil; UV (CH_3_OH) λ_max_ 310 (ε 6280), 229 (ε 3980) nm; ^1^H- and ^13^C-NMR data, see Table 1 and Table 2; HREIMS *m/z* 194.0938 [M]^+^ (calcd for C_11_H_14_O_3_, 194.0943).

### 3.4. Cytotoxicity Testing

The cancer cell lines CaSki and MDA-MB-231 were obtained from the Shanghai Institute of Cell Biology, Chinese Academy of Science, Shanghai, China. All cells were maintained in RPMI-1640 medium supplemented with 10% fetal calf serum, 25 mM HEPES buffer, 2 mmol/L L-glutamine, 100 U/mL penicillin and 100 μg/mL streptomycin. Cultures were incubated in a humidified atmosphere of 5% CO_2_ at 37 °C. Cells (1 × 10^4^/well) were seeded in supplemented culture medium (100 μL/well) in a 96-well plate and incubated for 24 h. The medium was then replaced with a test compound-containing medium, and the cells were further incubated for 48 h. All experiments were run in parallel with controls (0.1% DMSO) and the cell viabilities were evaluated by MTT assays. The absorbance of formazan formed was measured at 570 nm by a microplate reader. Each experiment was repeated three times.

## 4. Conclusions

Pestalafuranones F–J (compounds **3**–**7**) were obtained by adding 3% NaCl to the potato dextrose medium used to grow *Nigrospora* sp*.* BM-2 in an effort to activate silent genes and induce unique biosynthetic pathways. To our best knowledge, only five of these compounds (pestalafuranones A–E) have been previously reported in the literature [7]. These compounds were structurally characterized as *α*,*β*-unsaturated *γ*-lactone unit with a propenyl and a *n*-butyl side chain attached to C-3 and C-4, respectively. The chemical diversity of pestalafuranones was attributable to structural change of side chains, such as hydroxylation, oxidation, reduction and dehydrogenation. In the cytotoxic testing, pestalafuranones A,B and F–J (compounds **1**–**7**) exhibited no cytotoxic activities against MDA-MB-231 and Caski cancer cell lines. Further bioassay evaluation of the compounds against fungi and other targets are on-going.

## Supplementary Materials

Supplementary materials can be accessed at: http://www.mdpi.com/1420-3049/19/1/819/s1.
