# Supplementary material for: Pestalafuranones F–J, Five New Furanone Analogues from the Endophytic Fungus Nigrospora sp. BM-2
Source: Molecules. 2014 Jan 10;19(1):819–25. doi: 10.3390/molecules19010819 (PMC6271264; doi:10.3390/molecules19010819)

Supplementary Materials

Figure S1. HREIMS spectrum of Pestalafuranone F (**3**).

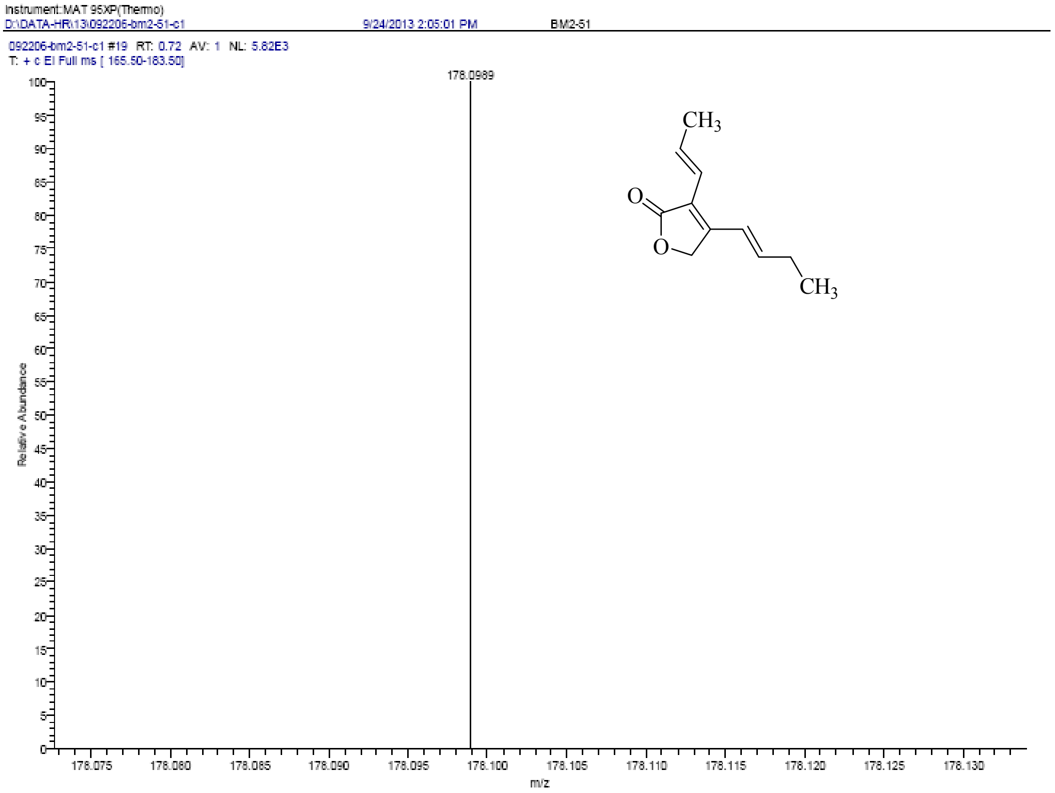

Figure S2. ^{1}H-NMR spectrum of Pestalafuranone F (**3**) (400 MHz, $CDCl_3$).

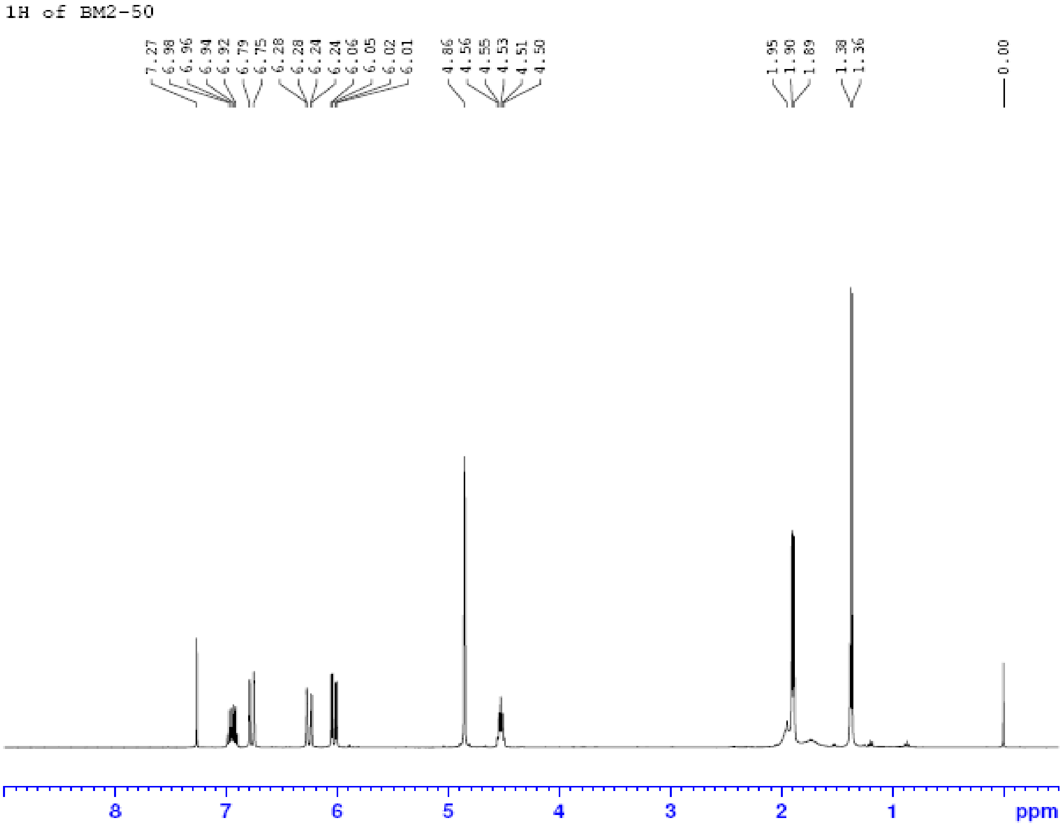

Figure S3. ^{13}C-NMR spectrum of Pestalafuranone F (**3**) (100 MHz, $CDCl_3$).

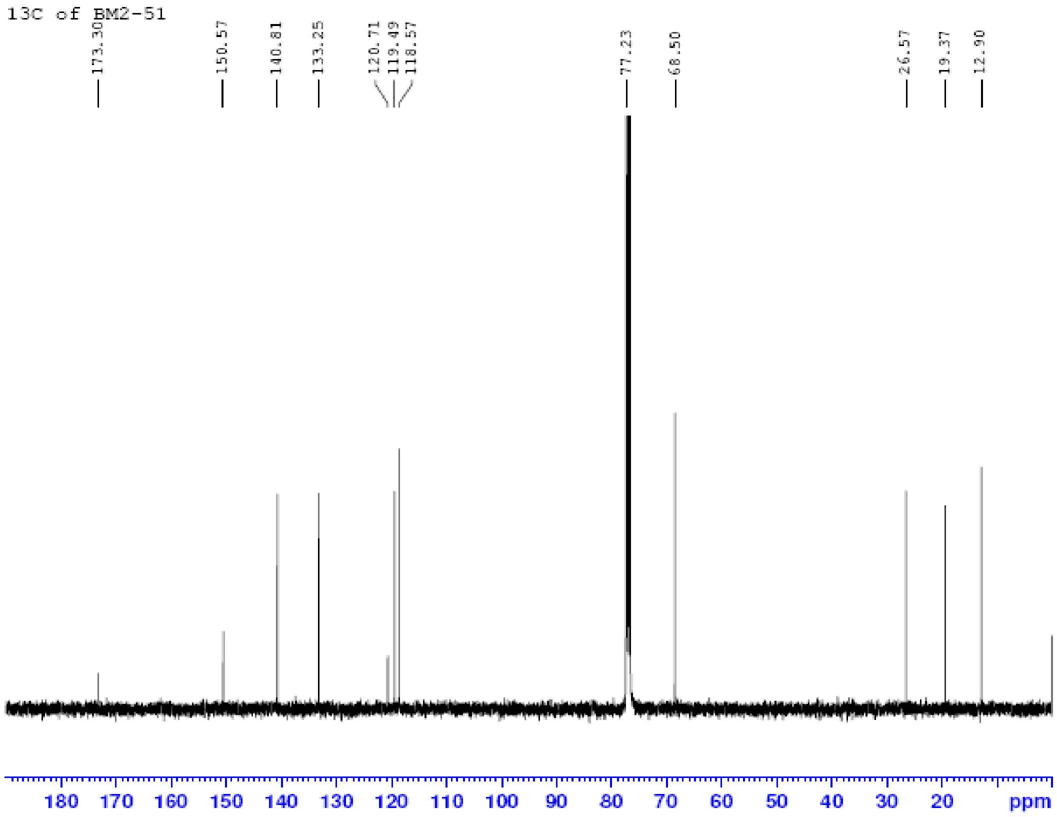

Figure S4. HREIMS spectrum of Pestalafuranone G (**4**).

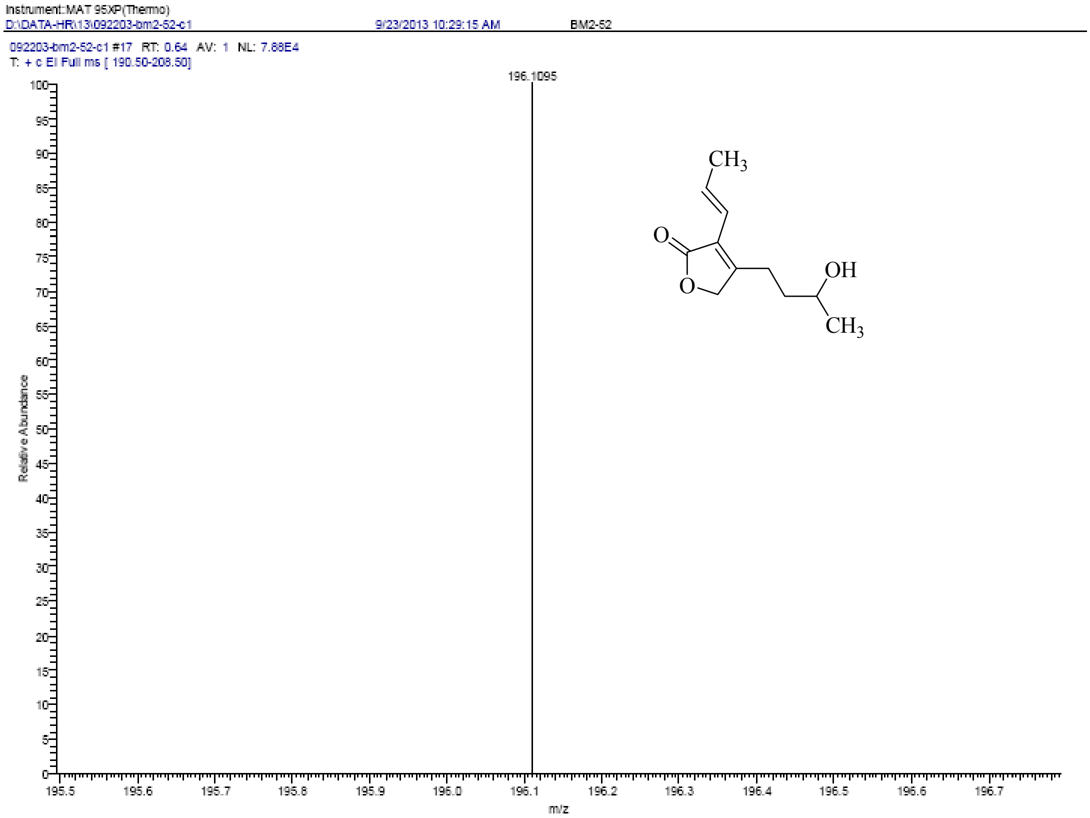

Figure S5. ^{1}H-NMR spectrum of Pestalafuranone G (**4**) (400 MHz, $CDCl_3$).

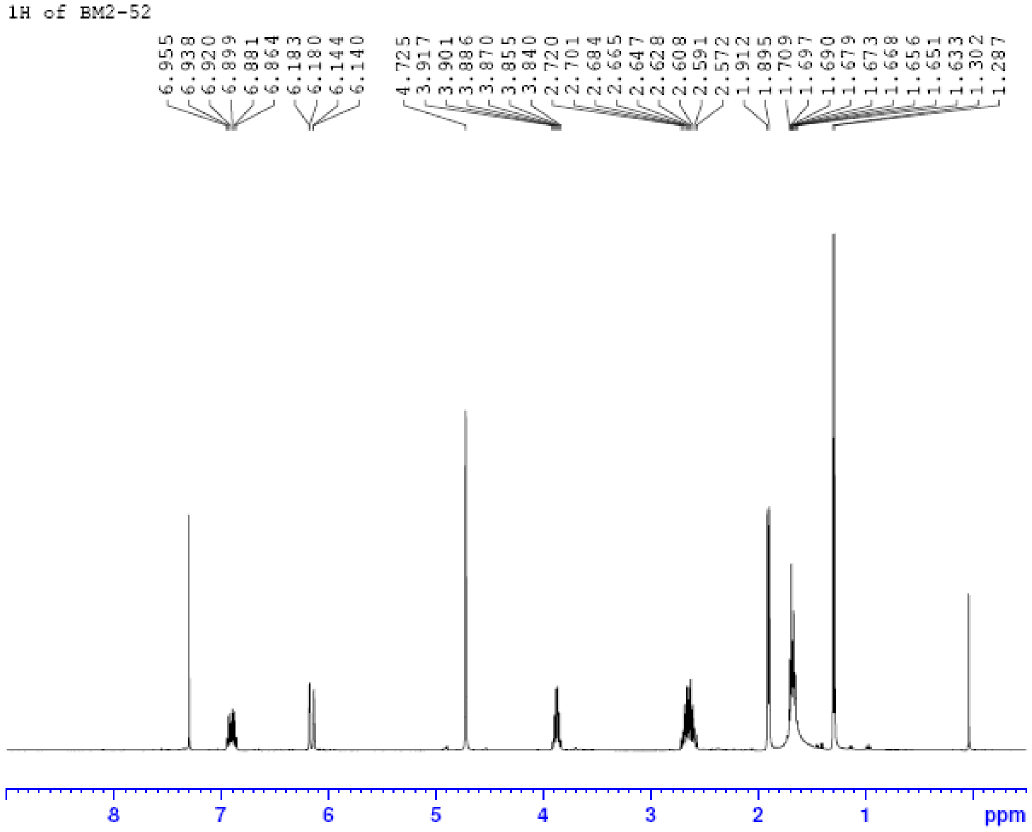

Figure S6. ^{13}C-NMR spectrum of Pestalafuranone G (**4**) (100 MHz, $CDCl_3$).

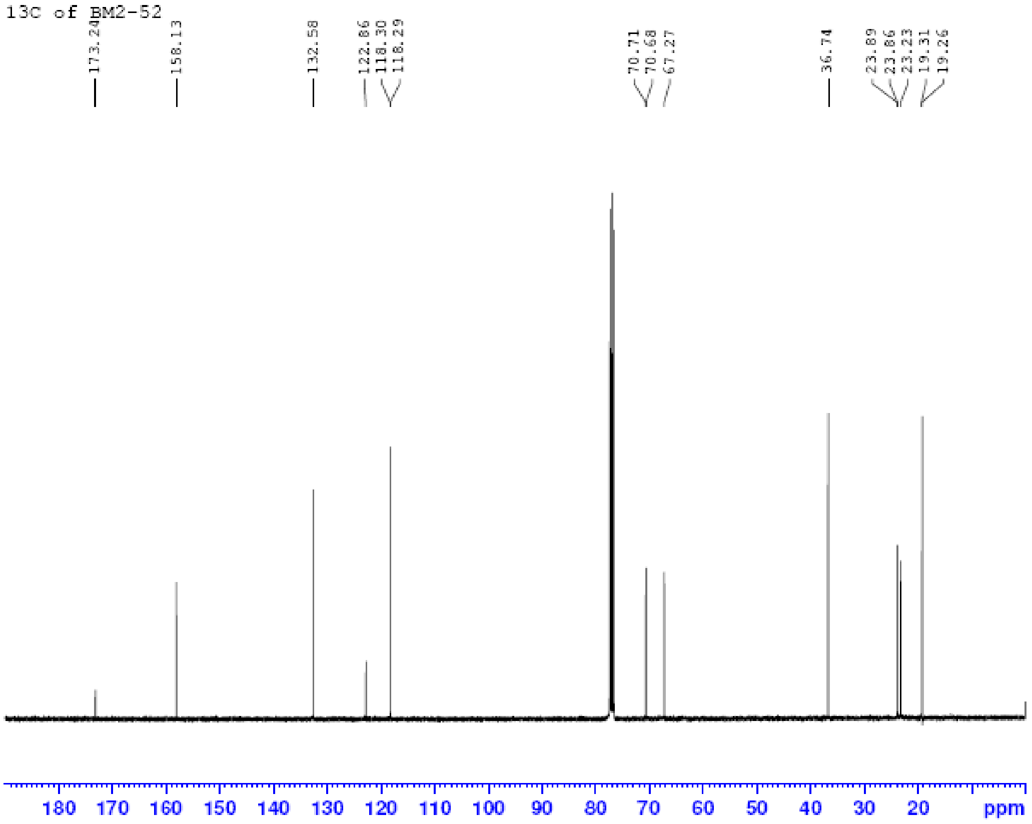

Figure S7. HREIMS spectrum of Pestalafuranone H (**5**).

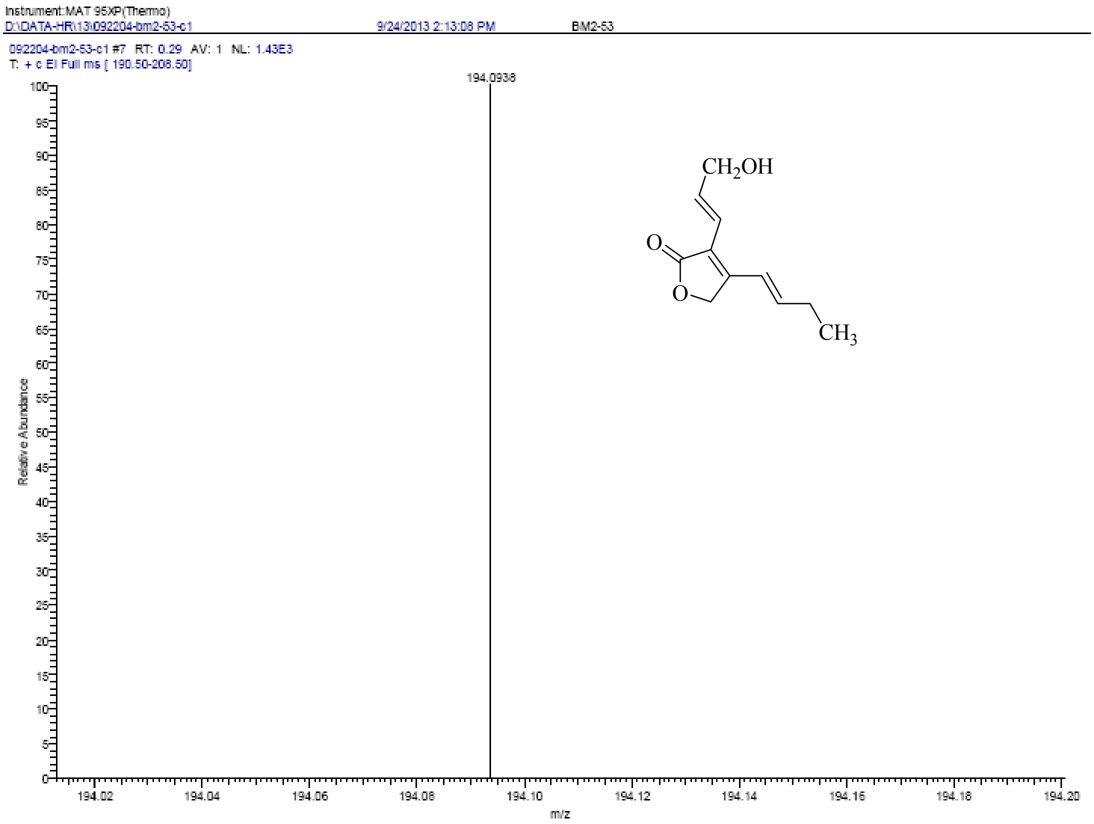

Figure S8. ^{1}H-NMR spectrum of Pestalafuranone H (**5**) (400 MHz, $CDCl_3$).

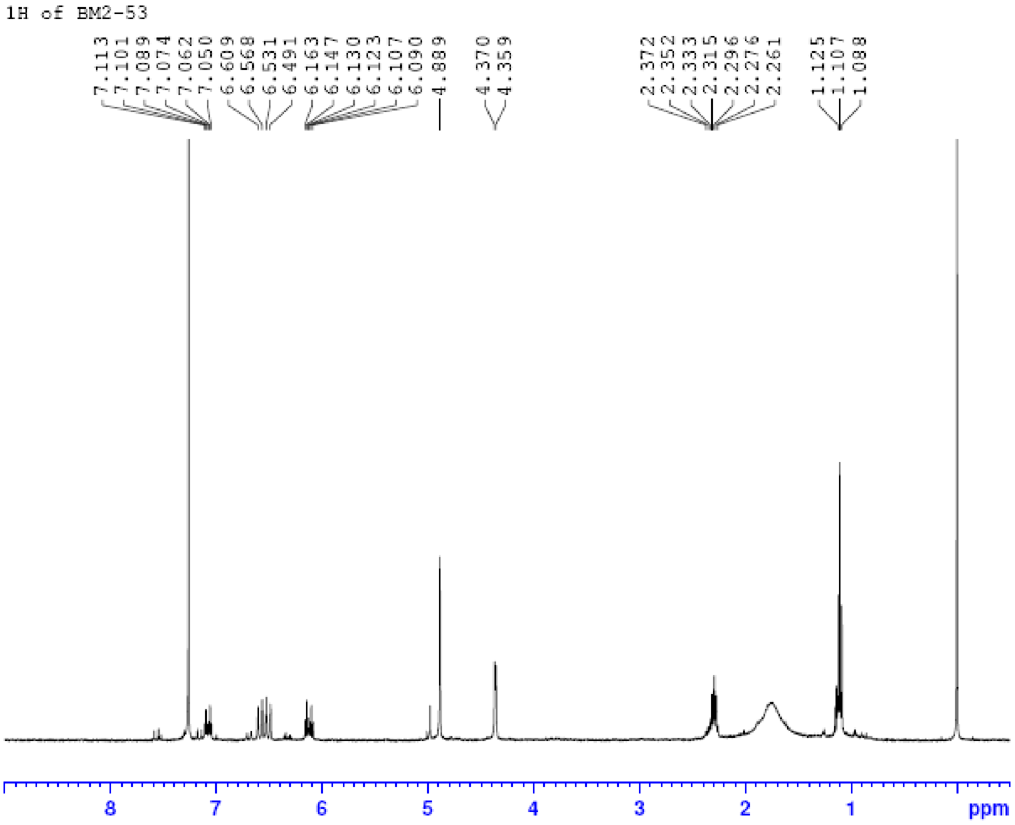

Figure S9. ^{13}C-NMR spectrum of Pestalafuranone H (**5**) (100 MHz, $CDCl_3$).

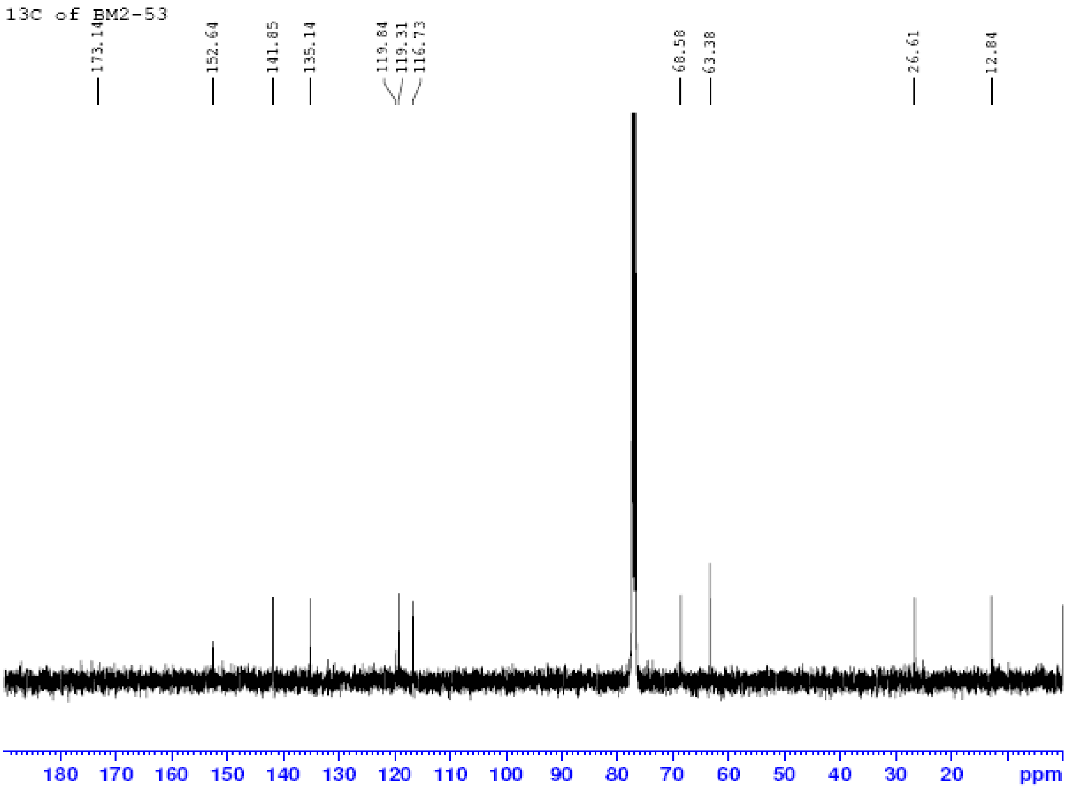

Figure S10. HREIMS spectrum of Pestalafuranone I (**6**).

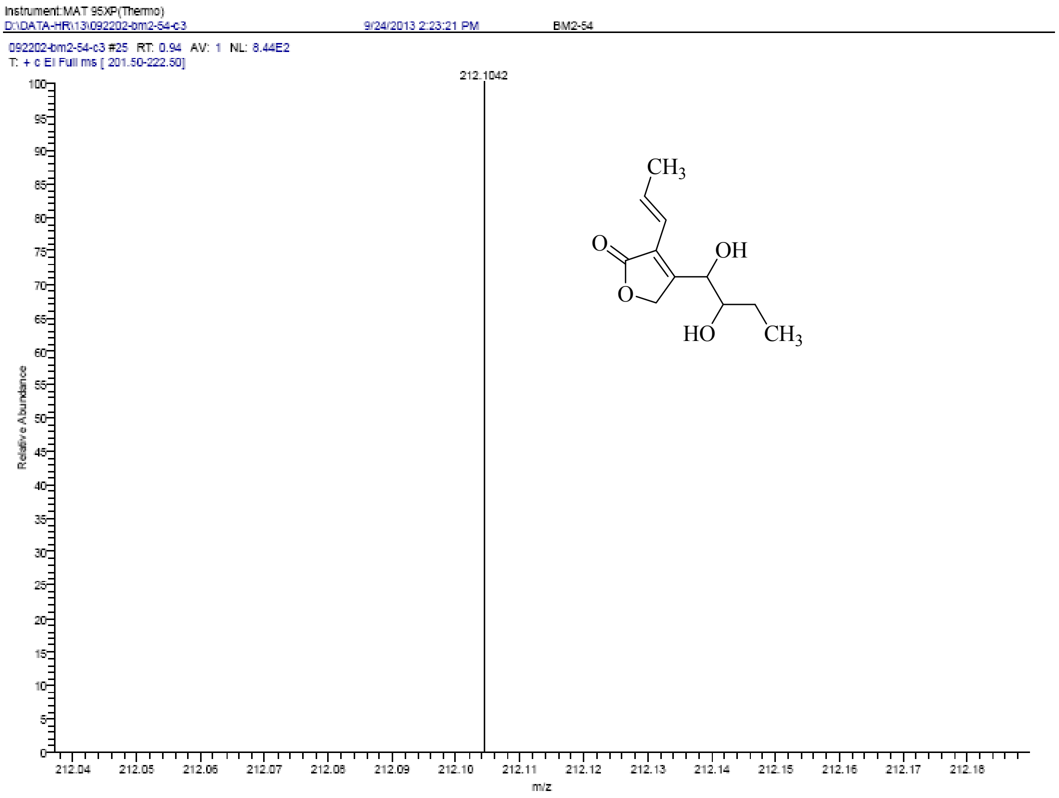

Figure S11. ^{1}H-NMR spectrum of Pestalafuranone I (**6**) (400 MHz, $CDCl_3$).

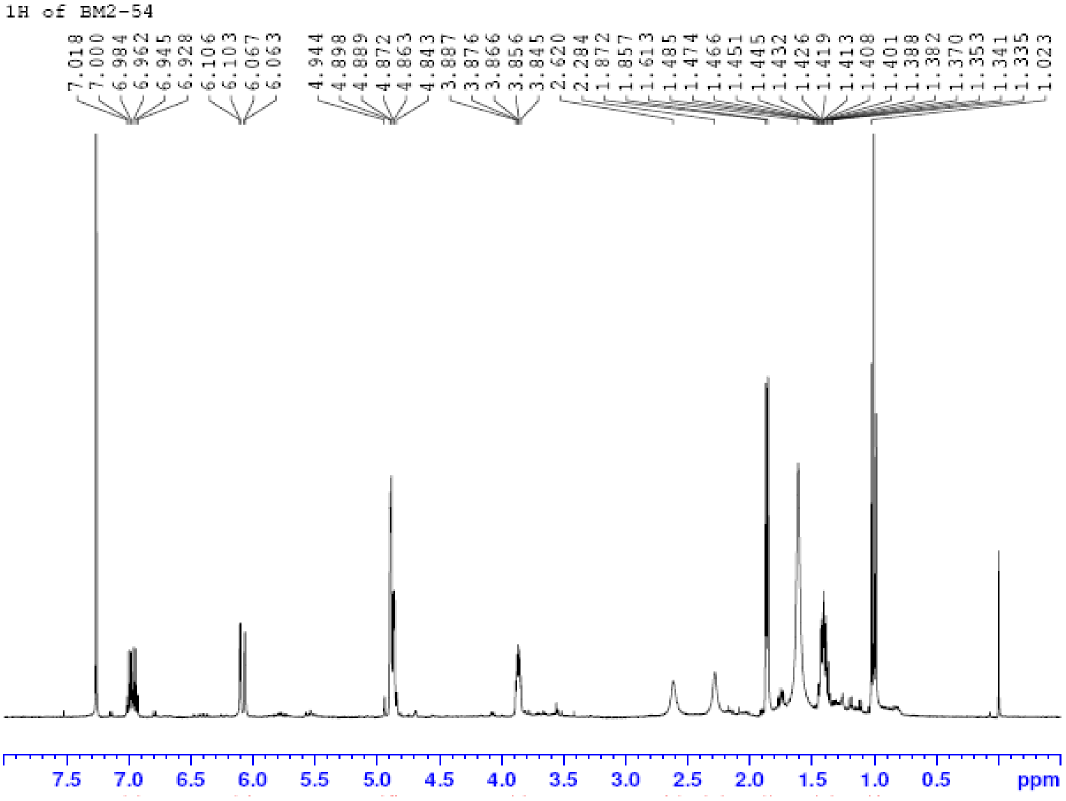

Figure S12. ^{13}C-NMR spectrum of Pestalafuranone I (**6**) (100 MHz, $CDCl_3$).

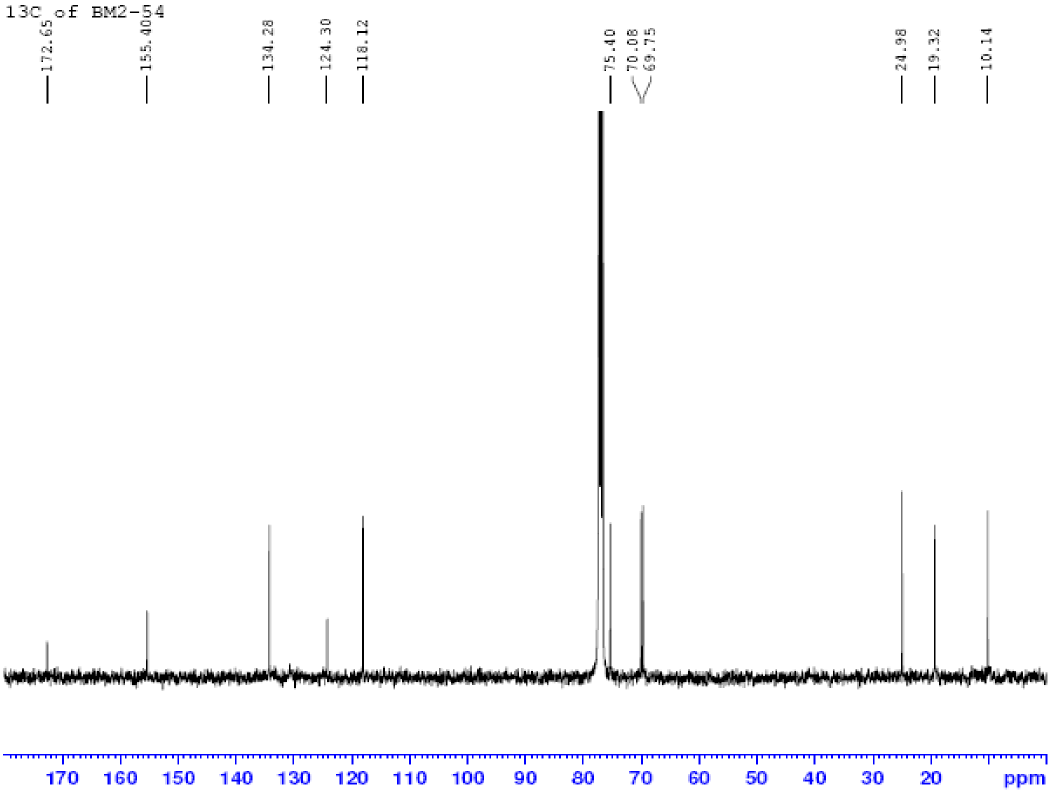

Figure S13. HREIMS spectrum of Pestalafuranone J (**7**).

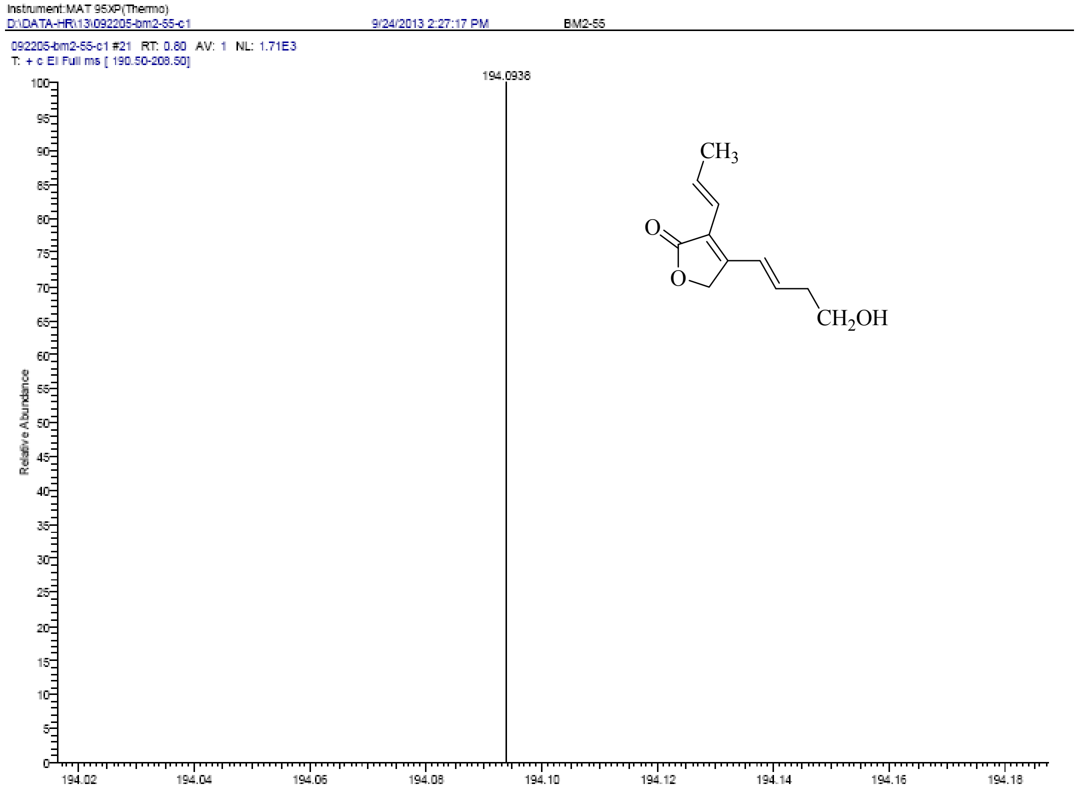

Figure S14. ^{1}H-NMR spectrum of Pestalafuranone J (**7**) (400 MHz, $CDCl_3$).

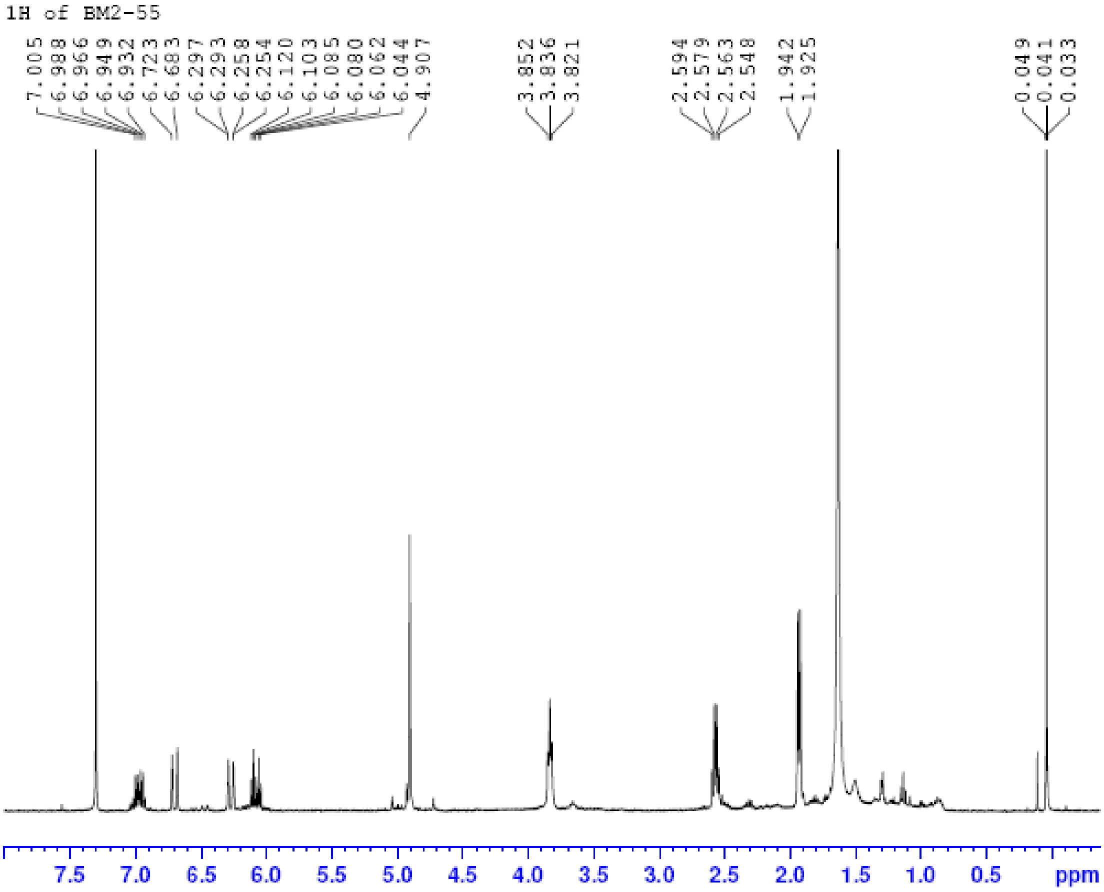

Figure S15. ^{13}C-NMR spectrum of Pestalafuranone J (**7**) (100 MHz, $CDCl_3$).

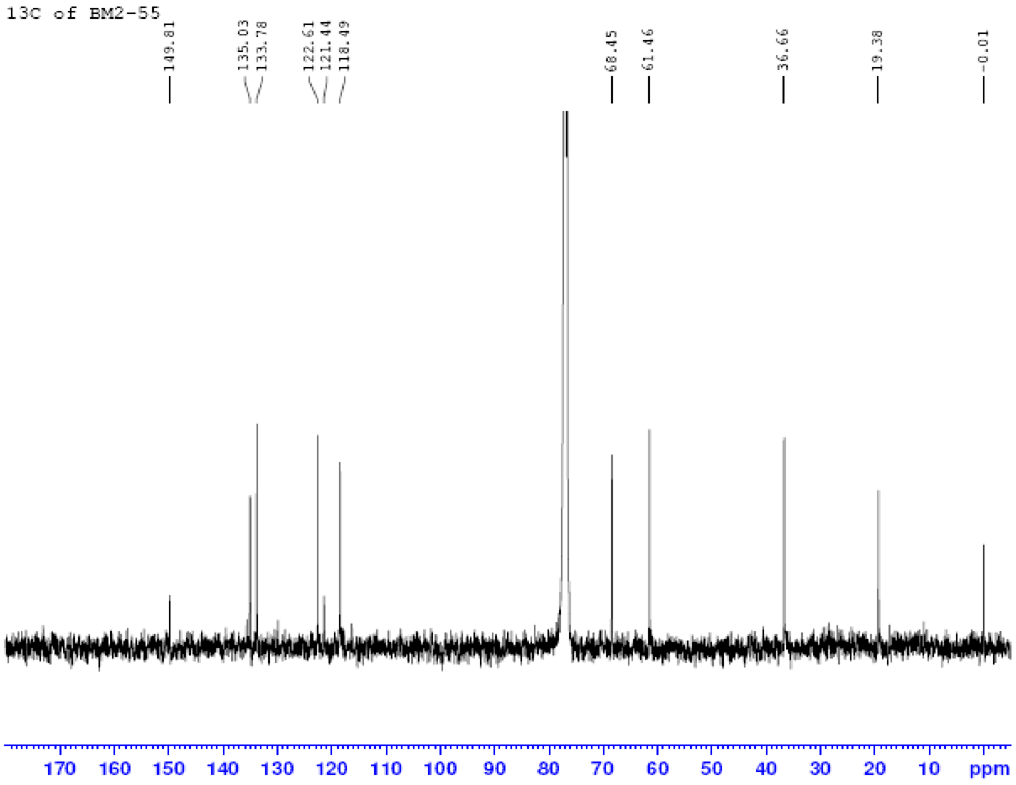

Supplement: Supplementary file 1 [file molecules-19-00819-s001.pdf]
